# Quantum–Classical Entropy Analysis for Nonlinearly-Coupled Continuous-Variable Bipartite Systems

**DOI:** 10.3390/e24020190

**Published:** 2022-01-27

**Authors:** Ángel S. Sanz

**Affiliations:** Department of Optics, Faculty of Physical Sciences, Universidad Complutense de Madrid, Pza. Ciencias 1, Ciudad Universitaria, 28040 Madrid, Spain; a.s.sanz@fis.ucm.es; Tel.: +34-91-394-4673

**Keywords:** Wigner distribution function, entanglement, quantum–classical correspondence, entropy measurement, quantum dynamics, quantum foundations, open quantum systems

## Abstract

The correspondence principle plays a fundamental role in quantum mechanics, which naturally leads us to inquire whether it is possible to find or determine close classical analogs of quantum states in phase space—a common meeting point to both classical and quantum density statistical descriptors. Here, this issue is tackled by investigating the behavior of classical analogs arising upon the removal of all interference traits displayed by the Wigner distribution functions associated with a given pure quantum state. Accordingly, the dynamical evolution of the linear and von Neumann entropies is numerically computed for a continuous-variable bipartite system, and compared with the corresponding classical counterparts, in the case of two quartic oscillators nonlinearly coupled under regular and chaos conditions. Three quantum states for the full system are considered: a Gaussian state, a cat state, and a Bell-type state. By comparing the quantum and classical entropy values, and particularly their trends, it is shown that, instead of entanglement production, such entropies rather provide us with information on the system (either quantum or classical) delocalization. This gradual loss of information translates into an increase in both the quantum and the classical realms, directly connected to the increase in the correlations between both parties’ degrees of freedom which, in the quantum case, is commonly related to the production of entanglement.

## 1. Introduction

Entanglement can be regarded as the characteristic trait of quantum mechanics, recalling Schrödinger [1], which means it plays a central role, as an essential resource, in the implementation and development of modern quantum technologies [2,3]. The capability to transfer information at long distances between two entangled parties without a physical interaction mediating between them [1,4] sets a crucial difference with respect to two classically correlated systems, where information transmission necessarily requires the active action of physical interactions. This is consistent with the common statement that there is no classical analog for entanglement. However, this seems in contradiction with the fact that, by virtue of the correspondence principle, classical mechanics should approximate quantum mechanics in one way or another, or, at least, manifest in some form. Thus, despite the uniqueness of entanglement as a genuine quantum trait, some relationships between the classically chaotic dynamics exhibited by nonlinear systems and entanglement have been found and reported in the literature. For instance, it has been found that the amount of entanglement increases in rates proportional to the corresponding Lyapunov exponents [5,6,7,8,9]. Indeed, this trend has also been considered the other way around, i.e., using the increase of entanglement as a signature of quantum chaos [5,9].

To further inquire about the issue, the common formal ground rendered by a Liouvillian or phase-space formulation, common to both classical and quantum mechanics, seems to be appropriate. In the quantum case, this is possible by describing the system state and its evolution within the Weyl–Wigner–Moyal representation [10,11,12]. In this representation, the density matrix $\hat{\rho }$ accounting for the system state in an *N*-dimensional configuration space is recast in terms of the corresponding Wigner quasi-distribution function [13]:

(1) $$\rho_{W}\left(r,p\right)=\frac{1}{{\left(\pi \hbar \right)}^{N}}\int \langle r-s|\hat{\rho }|r+s\rangle e^{2ip\cdot s/\hbar }ds,$$

where (r,p) is a 2N phase-space vector, with $r=(r_{1},r_{2},\ldots ,r_{N})$ and $p=(p_{1},p_{2},\ldots ,p_{N})$ being, respectively, the position and momentum vectors in such phase space. An advantage of this nonlinear representation is that it allows us to pass from a $C^{N}$ complex space to a $R^{2N}$ real space to describe the quantum system. This is, however, at the expense of translating interference traits, the distinctive signature of quantum coherence, into negative-definite regions in the associated phase space. The time evolution for $\rho_{W}$ is described by the Moyal equation

(2) $$\frac{\partial \rho_{W}\left(r,p,t\right)}{\partial t}=-\left\{\left\{\rho_{W}\left(r,p,t\right),H\left(r,p,t\right)\right\}\right\}=-{\left\{\rho_{W}\left(r,p,t\right),H\left(r,p,t\right)\right\}}_{P}+\mathrm{HOC}\left(\hbar^{2}\right),$$

where {{·,·}} is the Moyal bracket, defined as

(3) $$\left\{\left\{\cdot ,\cdot \right\}\right\}\equiv \frac{2}{\hbar }\sin\left[\frac{\hbar }{2}\left({\overset{\leftarrow }{\nabla }}_{r}{\vec{\nabla }}_{p}-{\overset{\leftarrow }{\nabla }}_{p}{\vec{\nabla }}_{r}\right)\right],$$

and ${\left\{\cdot ,\cdot \right\}}_{P}$ is the usual Poisson bracket, which arises from the lowest order of the Taylor expansion of the Moyal bracket, Equation (3). The term $\mathrm{HOC}(\hbar^{2})$ gathers all higher-order contributions of such Taylor expansion, which only includes explicit even powers of *ħ* (other than a possible implicit dependence on this parameter in the Wigner distribution function).

Formally, quantum–classical correspondence relies on the presence of the above HOC term. To better appreciate this statement, consider that the (phase space) Hamiltonian has the usual form $H\left(r,p\right)=p^{2}/2m+V\left(r\right)$. In this case, the HOC term reads as

(4) $$O\left(\hbar^{2}\right)=\sum_{n\geq 1}{\left(\frac{\hbar }{2}\right)}^{2n}\frac{{\left(-1\right)}^{n}}{(2n+1)!}\;\nabla_{r}^{2n+1}V\left(r\right)\;\nabla_{p}^{2n+1}\rho_{W}\left(r,p,t\right).$$

Accordingly, the quantum dynamics will resemble classical dynamical behaviors whenever this term becomes meaningless, e.g., whenever ħ→0, or if the potential is a polynomial of degree two or smaller. This is the reason why coherent (Glauber) states, for instance, can be regarded as classical states. If, on the other hand, the spatial variations of the potential function V(r) are relevant (higher than the second order), as it is the case of nonlinear potentials, particularly those inducing classically chaotic dynamics, the behavior of the Wigner distribution function will quickly develop typical quantum features, e.g., extensive negative-definite regions associated with the appearance of interference traits. However, this does not prevent the quantum system to be somehow influenced by an underlying classical dynamics mediated by the first term in the r.h.s. of Equation (2). This would explain, for instance, the above-mentioned relationship between quantum dynamics and (classical) Lyapunov exponents.

Nonetheless, there is also the possibility to observe important quantum effects with a vanishing HOC term. Think, for instance, of a simple superposition of two Gaussian wave packets. In this case, the direct analogy between Equation (2) and its classical counterpart breaks down immediately, even though the evolution equation is exactly the same in both cases. This is only as, in the quantum case, we allow the phase-space distribution (or quasi-distribution, strictly speaking) to be negative-definite in certain space regions, while classically we do not, which, on the other hand, is necessary in order to preserve the unitarity of $\mathrm{Tr}[{\hat{\rho }}^{2}]$ and hence the purity of the quantum state. Now, note that only in the case of (classical) Gaussian density distributions that preserve their Gaussianity all the way through the time evolution (i.e., in those cases where the potential function is a polynomial of degree two or lesser), the unitarity is preserved. This does not mean that a quantum and a classical Gaussian distribution are equivalent. Due to coherence preservation, the former is able to exhibit interference if it is superimposed with another Gaussian. This is not possible, though, in the classical case, because the corresponding distribution ($\rho_{\mathrm{cl}}$) makes reference to an incoherent swarm of particles, i.e., particles that have nothing to do one with the others. However, some works in the literature have dealt with the issue of determining classical-type eigendistributions in the Liouville phase space, analogous to the Wigner eigendistributions in the case of the quantum harmonic oscillator [14,15,16] and also in the case of the evolution of anharmonic oscillators [17].

Now, in general, $\mathrm{Tr}[\rho_{\mathrm{cl}}^{2}]\leq 1$, which considers the case of any arbitrary classical distribution, and follows from the Liouville theorem. The issue has been investigated earlier on in the context of quantum–classical correspondence for decoherence induced by nonlinear couplings in bipartite systems [18,19,20]. The elementary tool to explore this correspondence is the computation of entropies associated with one of the two subsystems, such as the linear and the von Neumann entropies. These entropies can be understood [3] as a measurement of entanglement produced over time by quantifying the degree of mixedness of one of the parties. Note that, as it was pointed out by Schrödinger [1], as soon as two systems start interacting, their state can no longer be described in a separate manner, regardless of whether they are spatially separated (typical EPR condition [4]) or not. An increase in the amount of entanglement means an increase in our lack of knowledge on the system state, which, in turn, translates into an increase in the corresponding entropy measure. This issue presents a remarkable similarity with former studies relating chaos with energy relaxation rates [21,22,23] or decoherence [24,25,26,27]. Furthermore, it is significant that entanglement dynamics of initially Gaussian states can be fully described by means of a classical entropy in the classical limit [28].

In order to evaluate how close classical distributions may approximate quantum ones in entropy measures, here we reexamine this problem considering two nonlinearly coupled one-dimensional systems, such that depending on the coupling strength the corresponding classical dynamics can be either regular or chaotic. In particular, we shall focus on the analysis of the time evolution exhibited by the entropy of one of the subsystems. This behavior will be analyzed not only in the case of Gaussian states, for which the initial classical counterpart is clear, but also with (superposition) cat states and Bell-type entangled states, which are non-classical states with interest both in quantum mechanics as well as in quantum optics [29,30,31,32,33]. In these cases, the classical analogs will be determined from the corresponding Wigner distribution functions, removing the negative-definite parts. This choice relies on the fact that, in the classical limit, the rapidly oscillatory behavior displayed by the Wigner distribution function generates negative-definite regions with negligible area. Indeed, as it is shown, in the classical limit, both the quantum system and its classical counterpart display analogous entropy rates. Accordingly, we provide an interpretation for the increase in the quantum entropy, which makes clear not only why it approaches the classical one, but also why it increases in the classical limit. This might seem counter-intuitive, as the linear and von Neumann entropies are supposed to be quantifiers of entanglement [34,35,36,37], which, on the other hand, has no classical counterpart. This behavior thus leads to an alternative manner to interpret these measures, as quantifiers of the maximum delocalization undergone by the system, a well-defined concept in both quantum and classical mechanics. Accordingly, due to the incoherence nature of the swarms of trajectories associated with classical distributions, it is seen that the classical entropies set an upper bound over the corresponding quantum entropies. The fact that the latter remain lower can be associated with a higher information compression in quantum mechanics, which is possible due to the coherence swapping between the two entangled parties.

The work is organized as follows. The specific functional form of the linear and von Neumann entropies in phase space is introduced in Section 2 for classically and quantum mechanically. Moreover, to be self-contained, some general properties are also discussed. The working model and corresponding numerical simulations carried out are presented and discussed in Section 3. The definitions of the classical analogs to the quantum states considered are also introduced in this section. These continuous-variable bipartite states include Gaussian (classical) states, cat states, and Bell-type entangled states. The general picture arising from the results described in Section 3 is provided in Section 4. To conclude, a series of summarizing remarks are given in Section 5.

## 2. Quantifying Entanglement in Phase Space

Different measurements have been proposed in the literature to quantify entanglement [34,35,36,37]. Among these entanglement quantifiers, the most commonly used ones for pure states are the linear and the von Neumann entropies [38,39,40,41]. For mixed states, other more general quantifiers have been proposed, such as the entanglement of formation [42], the entanglement of distillation [43,44], or the negativity [45,46,47,48]. Here, given that only pure states are considered to describe the full or joint system, the use of linear and von Neumann entropies suffices to carry out the quantum–classical analysis. Furthermore, because the joint state remains pure along time, the amount of entanglement of both parties is the same at any time (which can easily be proven using the Schmidt decomposition), thus ensuring that it can be measured with respect to any of the two subsystems.

Thus, let us assume that *X* and *Y* represent two one-dimensional continuous-variable systems, specified by their positions *x* and *y*, respectively. Without any loss of generality, the subsystem *X* will be taken as the reference system, and *Y* as the environment, which will be traced out. The reduced density matrix describing the state of *X* is ${\tilde{\rho }}_{X}={\mathrm{Tr}}_{Y}\left(\hat{\rho }\right)$, where $\hat{\rho }$ is the density matrix accounting for the XY joint system. Accordingly, the linear entropy is defined as

(5) $$S_{L}\equiv 1-{\mathrm{Tr}}_{X}\left({\tilde{\rho }}_{X}^{2}\right).$$

This is a direct measure of the loss of purity—determined by the quantity ${\mathrm{Tr}}_{X}\left({\tilde{\rho }}_{X}^{2}\right)$—undergone by *X* as it becomes more entangled with *Y* due to the interaction, i.e., as its degree of mixedness (classicality) increases. For a pure state, given that its purity is 1, the linear entropy vanishes. Note that the idempotence property of the density matrix is preserved in this case, so ${\tilde{\rho }}_{X}^{2}={\tilde{\rho }}_{X}$ and, consequently, ${\mathrm{Tr}}_{X}\left({\tilde{\rho }}_{X}^{2}\right)={\mathrm{Tr}}_{X}\left({\tilde{\rho }}_{X}\right)=1$. For a mixed state, however, this property gets lost, and ${\mathrm{Tr}}_{X}\left({\tilde{\rho }}_{X}^{2}\right)<{\mathrm{Tr}}_{X}\left({\tilde{\rho }}_{X}\right)=1$. Thus, in sum, we have

(6) $$0\leq S_{L}\leq 1,$$

where the upper bound is approached as the degree of mixedness increases. An evident advantage of this quantity is its relative simplicity to provide us with information about the amount of entanglement between the two parties by observing how one of them gradually losses its purity (i.e., as the action of decoherence becomes more relevant). This has been beneficially used, for instance, in studies relating chaotic dynamics with production of entanglement [6,7,8].

In the Wigner representation, Equation (5) reads as

(7) $$S_{L}=1-2\pi \hbar \int {\tilde{\rho }}_{W}^{2}\left(x,p_{x}\right)dxdp_{x},$$

where

(8) $${\tilde{\rho }}_{W}\left(x,p_{x}\right)={\mathrm{Tr}}_{Y}\left[\rho_{W}\left(x,y,p_{x},p_{y}\right)\right]=\int \rho_{W}\left(x,y,p_{x},p_{y}\right)dydp_{y}.$$

If the Wigner distribution function provides us with a phase-space picture of the quantum system, analogously in classical statistical mechanics we have density distributions accounting for the statistical distribution of phase-space trajectories. Therefore, we can introduce the classical analog of Equation (7) as

(9) $$S_{L}^{\mathrm{cl}}=1-2\pi \hbar \int {\tilde{\rho }}_{\mathrm{cl}}^{2}\left(x,p_{x}\right)dxdp_{x},$$

where ${\tilde{\rho }}_{\mathrm{cl}}\left(x,p_{x}\right)$ specifies a classical density distribution in the reduced phase subspace associated with the reference system *X*, obtained after integrating over all other phase-space coordinates (those related to *Y*, in this case).

Regarding the von Neumann entropy, it is a generalization of the Shannon entropy used in classical information theory [49,50], and is defined as

(10) $$S_{V}\equiv -{\mathrm{Tr}}_{X}\left[{\tilde{\rho }}_{X}\ln{\tilde{\rho }}_{X}\right]=-\sum_{i}\lambda_{i}\log\lambda_{i},$$

where $\lambda_{i}$ denotes the eigenvalues of the reduced density matrix. This quantity determines the amount of information that is stored in the quantum state describing the system of reference, which increases with its degree of mixture, and is considered to be the best measure of entanglement for pure states [41]. Apart from its connection to thermodynamics [41], this entropy measure has also some properties that make it of interest in quantum statistical mechanics and quantum information theory [3,38,39,40]. Note that, despite their differences, the linear and von Neumann entropies are going to show a similar trend as the quantum system evolves, which is also expected in the case of their classical counterparts if there is a proper correspondence. Furthermore, although the linear entropy is not an approximation of the von Neumann entropy, it can readily be seen that, by Taylor expanding the latter, the first two terms correspond to the former:

(11) $$S_{V}=-\mathrm{Tr}\left(\tilde{\rho }\ln\tilde{\rho }\right)=-\mathrm{Tr}\left[\tilde{\rho }\sum_{k=1}^{\infty }{\left(-1\right)}^{k+1}\;\frac{{\left(\tilde{\rho }-I\right)}^{k}}{k}\right]=1-\mathrm{Tr}\left({\tilde{\rho }}^{2}\right)-\mathrm{Tr}\left[\tilde{\rho }\sum_{k=2}^{\infty }{\left(-1\right)}^{k+1}\;\frac{{\left(\tilde{\rho }-I\right)}^{k}}{k}\right].$$

This thus shows that the von Neumann entropy partly contains basic information about the system mixedness also provided by the linear entropy.

In the Wigner representation, the von Neumann entropy reads as

(12) $$S_{V}=-2\pi \hbar \int {\tilde{\rho }}_{W}\left(x,p_{x}\right){\left(\ln\tilde{\rho }\right)}_{W}\left(x,p_{x}\right)dxdp_{x}.$$

Note that, due to the non-commutativity of the logarithm operation and the Wigner transform, ${\left(\ln\tilde{\rho }\right)}_{W}\neq \ln{\tilde{\rho }}_{W}$. This avoids the drawback of the negativity displayed by the Wigner distribution function ${\tilde{\rho }}_{W}$ in phase-space regions associated with interference. In the classical case, as distributions are positive definite on any phase-space region, it can be assumed that the equality holds. Thus, we consider here the classical analog of (12) as a Shannon-type entropy, henceforth denoted as $S_{V}^{\mathrm{cl}}$, with functional form

(13) $$S_{V}^{\mathrm{cl}}=-\int {\tilde{\rho }}_{\mathrm{cl}}\left(x,p_{x}\right)\ln\left[2\pi \hbar {\tilde{\rho }}_{\mathrm{cl}}\left(x,p_{x}\right)\right]dxdp_{x}.$$

Note in this expression the 2πħ factor inside the logarithm, necessary to obtain a dimensionless argument. In the quantum expression, Equation (12), it is outside the logarithm, because the density matrix is dimensionless; in this case, the action dimensions are assigned to the full function ${\left(\ln\tilde{\rho }\right)}_{W}$. Consequently, although the quantum and classical expressions for the linear entropy keep a close correspondence, the same does not hold for the von Neumann entropy, which is directly connected to the third term on the r.h.s. of the second equality in Equation (11). Hence, for instance, in the case of quantum and classical states described in phase space by a Gaussian distribution, the linear entropy is zero in both cases, while the von Neumann entropy only vanishes for the quantum state; the “classical” von Neumann entropy renders a value of 0.307 (see Section 3.2).

## 3. Results and Discussion

### 3.1. Working System and Dynamical Evolution

The working system here consists of two identical one-dimensional quartic oscillators coupled by a nonlinear term, described by the Hamiltonian

(14) $$H\left(x,y,p_{x},p_{y}\right)=\frac{p_{x}^{2}}{2m}+\frac{p_{y}^{2}}{2m}+\frac{\beta }{4}\left(x^{4}+y^{4}\right)+\frac{\alpha }{2}\;x^{2}y^{2}.$$

**Figure 1:**
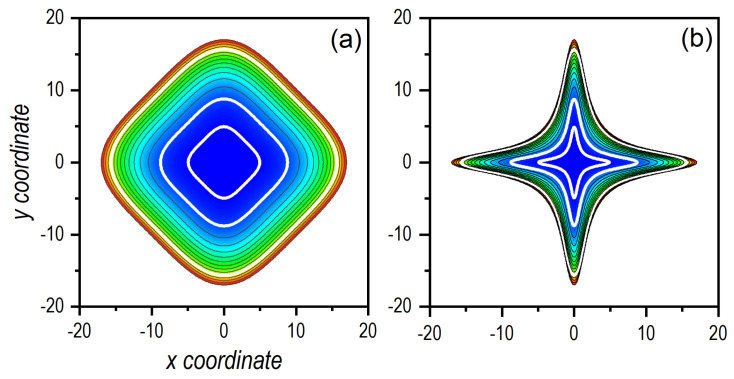
Density plots of the quartic potential for β=0.01 and two values of α that generate different dynamics: (**a**) α=0.03 (regular dynamics) and (**b**) α=1 (chaotic dynamics). From blue to red, increasing value of the potential function, black contours indicate equipotential lines at multiples of E=15 (last contour shown corresponds to E=210). White contours in both panels represent the equipotential lines for $E_{0}=1.5,15$, and 150, which are the energies considered in the numerical simulations here (see next sections).

Due to its connection to chaotic dynamics, this model has been extensively studied in the literature both classically [51,52,53] and also quantum mechanically [53,54,55,56]. In particular, the following parameter values have been considered: m=1, β=0.01, and, for the quantum case, ħ=1, all in arbitrary units. Regarding the α parameter, which controls the dynamics, two values have been chosen, namely, α=0.03, which produces regular dynamics, and α=1, which leads to fully chaotic dynamics. The two cases are illustrated in the density plots displayed in Figure 1, where black solid lines denote contours separated by multiples of 15 energy units, from 0 to a maximum energy value of 210. The white solid contours denote the three energies considered in the calculations described in next sections $E_{0}=1.5,15$, and 150. As it is clearly seen, changing from α=0.03 to α=1 leads to a dramatic change of the equipotentials, which translates into passing from regular dynamical regimes to chaotic ones.

The numerical simulations have been carried out as follows. Quantum mechanically, the split-operator algorithm has been used to solve the time-dependent Schrödinger equation [57]. At each time-step, the density matrix has been computed, its trace over the subsystem *Y* has been performed (integrating over the *y*-coordinate), and then the reduced density matrix for the subsystem *X* has been numerically diagonalized, from which both the linear and von Neumann entropies have been obtained. Note that, from a practical point of view, there is no need to formerly determine the Wigner function, because the interest relies directly on the actual values of the entropy and not on the explicit form of the corresponding Wigner distribution function, thus being independent of the representation considered. This procedure thus avoids an unnecessarily extra increase in the computational demand involved in the calculation of the four-dimensional Wigner distribution function for the full system. As for the choice of the initial states, they are described in more detail in the next sections, in the context of the actual computations. In all cases considered, i.e., Gaussian states, cat states, and Bell-type entangled states, the width of the corresponding wave packets involved has been chosen to be $\sigma^{2}=0.5$.

Concerning the classical calculations, they are based on performing statistics over trajectories that obey the Hamilton equations of motion corresponding to the Hamiltonian (14), with their initial conditions selected according to the corresponding classical distribution (see next sections), thus corresponding to an average energy around a certain value $E_{0}$. As the potential function is homogeneous, the classical dynamics is energy-scale invariant, i.e., for α and β fixed, the same dynamics is observed at any value of $E_{0}$. However, the statistics change, because the size and shape of the initial distribution of the swarm of trajectories also play a role. Moreover, the scale-invariance does not apply either to the quantum dynamics, where the larger $E_{0}$, the larger the number of eigenstates involved in the dynamics [56]. The statistics have been carried out over a total of $10^{6}$ trajectories by means of a box-counting procedure, which renders a fair coarse-grained value of the corresponding entropies [21,22,23]. In fact, to establish a better comparison between the classical and quantum results, the pixel size of the reduced phase space (i.e., the phase space for the *X* subsystem, determined by *x* and $p_{x}$), such box-counting has been performed taking into account the grid size associated with the quantum partner. Once the classical distribution is reconstructed at each time, in terms of a discretized phase space, the classical linear and von Neumann entropies are straightforwardly computed by also discretizing the corresponding trace operations (i.e., summing up over pixels).

### 3.2. Gaussian State

Let us first consider that both subsystems are initially described by localized Gaussian states in the phase space, around the points $\left(x_{0},p_{x,0}\right)$ and $\left(y_{0},p_{y,0}\right)$, such that they are bound by the energy conservation condition

(15) $$E_{0}=\frac{p_{x,0}^{2}}{2m}+\frac{p_{y,0}^{2}}{2m}+\frac{\beta }{4}\left(x_{0}^{4}+y_{0}^{4}\right)+\frac{\alpha }{2}\;x_{0}^{2}y_{0}^{2}.$$

The joint state is given by the separable wave function

(16) $$\Psi \left(x,y\right)={\left(\frac{1}{2\pi \sigma^{2}}\right)}^{1/2}e^{-{\left(x-x_{0}\right)}^{2}/4\sigma^{2}+ip_{x,0}\;\;x/\hbar }e^{-{\left(y-y_{0}\right)}^{2}/4\sigma^{2}+ip_{y,0}\;\;y/\hbar }.$$

Due to its factorizability, the corresponding Wigner distribution function is simply the product of the reduced Wigner distribution functions associated with each subsystem:

(17) $$\rho_{W}\left(x,y,p_{x},p_{y}\right)={\left(\frac{1}{\pi \hbar }\right)}^{2}e^{-{\left(x-x_{0}\right)}^{2}/2\sigma^{2}-2\sigma^{2}{\left(p_{x}-p_{x,0}\right)}^{2}/\hbar^{2}}e^{-{\left(y-y_{0}\right)}^{2}/2\sigma^{2}-2\sigma^{2}{\left(p_{y}-p_{y,0}\right)}^{2}/\hbar^{2}}.$$

Thus, tracing over *Y* has no influence on the reduced Wigner distribution function for *X*, which reads as

(18) $${\tilde{\rho }}_{W}\left(x,p_{x}\right)=\left(\frac{1}{\pi \hbar }\right)e^{-{\left(x-x_{0}\right)}^{2}/2\sigma^{2}-2\sigma^{2}{\left(p_{x}-p_{x,0}\right)}^{2}/\hbar^{2}}.$$

From this expression, it is readily shown that the purity satisfies the above-mentioned unitarity property, i.e.,

(19) $${\mathrm{Tr}}_{X}\left[{\tilde{\rho }}_{W}^{2}\right]=2\pi \hbar \int {\tilde{\rho }}_{W}^{2}\left(x,p_{x}\right)dxdp_{x}=1,$$

and hence the linear entropy, $S_{L}$, vanishes. This result is valid for both the quantum and the classical case. The same, though, does not hold for the von Neumann entropy, which vanishes in the quantum case, but its classical counterpart, Equation (13), renders

(20) $$S_{V}^{\mathrm{cl}}=1-\ln2\approx 0.307.$$

This value constitutes a lower bound for the classical von Neumann entropy; at any other stage of its evolution, governed by the Hamiltonian (14), the system entropy will be larger regardless of the dynamics exhibited (either regular or chaotic). This lower bound for the classical von Neumann entropy can be associated with the lack of coherence of classical distributions, which not only prevents them from developing and displaying interference traits, but also to compress and minimize the amount of phase-space information contained in them. In other words, while a quantum pure state provides us with full information about the system (we cannot further specify the state beyond the information encoded in its density matrix), classically a statistical sampling of incoherent phase-space points (swarm of independent trajectories) is required to determine the system state.

**Figure 2:**
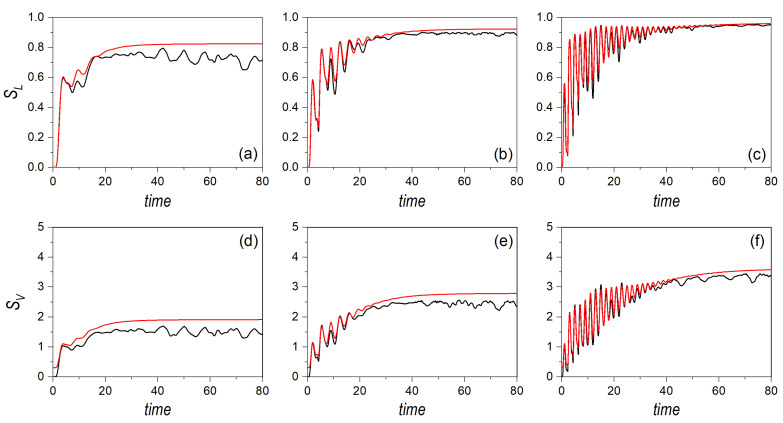
Linear entropy (upper row panels) and von Neumann entropy (lower row panels) for an initial distribution associated with a single Gaussian wave packet at three different energies: $E_{0}=1.5$ (**a**,**d**), $E_{0}=15$ (**b**,**e**), and $E_{0}=150$ (**c**,**f**). The dynamics corresponds to regularity conditions, with α=0.03. In all panels, the black line denotes the quantum results, while the red line represents the classical ones.

**Figure 3:**
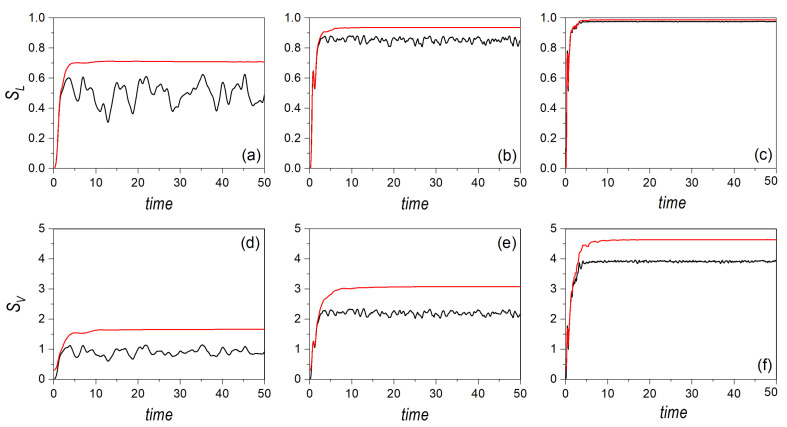
Linear entropy (upper row panels) and von Neumann entropy (lower row panels) for an initial distribution associated with a single Gaussian wave packet at three different energies: $E_{0}=1.5$ (**a**,**d**), $E_{0}=15$ (**b**,**e**), and $E_{0}=150$ (**c**,**f**). The dynamics corresponds to chaos conditions, with α=1. In all panels, the black line denotes the quantum results, while the red line represents the classical ones.

If the Gaussian distributions are left to evolve according to the dynamics ruled by the Hamiltonian (14), we find that the above analytical results are in agreement with the numerical simulations at the early stages of the evolution, as can be seen in all cases represented in Figure 2 and Figure 3. In these figures, the linear entropy is shown in the upper row panels, while the von Neumann entropy is displayed in the lower row ones for α=0.03 (Figure 2) and α=1 (Figure 3). In all cases, the phase-space point around which the Gaussian distribution is centered corresponds to $\left(0,0,\sqrt{mE_{0}},\sqrt{mE_{0}}\right)$, i.e., the periodic orbit moving along the diagonal y=x [54], with $E_{0}=1.5$ (a/d), $E_{0}=15$ (b/e), and $E_{0}=150$ (c/f). In Figure 2, it can be noticed in that, under regular conditions, the classical entropies follow very closely the behavior displayed by their quantum counterparts, with a better agreement, indeed, at larger energies, where the amount of eigenstates involved in the quantum dynamics is larger. Actually, the oscillations (recurrences) undergone by both entropies are nicely followed by the classical distribution, even though one might expect important interference-mediated differences. This trend is more clearly seen (for a longer time) as the frequency of the periodic orbit becomes larger, which happens as $E_{0}$ increases. A rather simple estimation suffices to show that, classically, the half-period, $\tau_{1/2}$, associated with these orbits is a function of $E_{0}$:

(21) $$\tau_{1/2}\left(E_{0}\right)=\frac{1}{\sqrt{mE_{0}}}\int_{x_{-}}^{x_{+}}\frac{dx}{\sqrt{1-\gamma x^{4}}}=\frac{2x_{+}}{\sqrt{mE_{0}}}\;{}_{2}F_{1}\left(1/4,1/2;5/4;x_{+}^{4}\gamma \right)\approx 3.12{\left[m^{2}\left(\beta +\alpha \right)E_{0}\right]}^{-1/4},$$

where $x_{\pm }=\pm \gamma^{-1/4}$ denote the turning points of the periodic orbit, with $\gamma =\left(\beta +\alpha \right)/2E_{0}$, and ${}_{2}F_{1}\left(a,b;c;z\right)$ is the Gaussian or ordinary hypergeometric function of the first kind, with ${}_{2}F_{1}\left(1/4,1/2;5/4;1\right)\approx 1.31$. The values of the half period corresponding to the three energies here considered are: $\tau_{1/2}\left(1.5\right)\approx 6.30$, $\tau_{1/2}\left(15\right)\approx 7.1$, and $\tau_{1/2}\left(150\right)\approx 1.99$. As the Gaussian distribution is launched from the center of the potential, it will undergo a recurrence at half the period, each time that the classical periodic orbit crosses this point (maxima indicate maximum amplitude in the coordinate, and minima, maximum amplitude in the momentum).

In Figure 2a, however, recurrences are hardly seen, which is due to the smearing out undergone by the distribution, as the back-and-forth oscillatory motion of the Gaussian is slower than its spreading. To better understand this effect, consider a free Gaussian wave packet. Its spreading in time is determined by the simple expression [57,58]

(22) $$\sigma \left(t\right)=\sigma \sqrt{1+{\left(\frac{\hbar t}{2m\sigma^{2}}\right)}^{2}}.$$

In the particular case of the bound potential here considered, we can assume that there is an upper bound for the time at which the width of the wave packet will cover the extension between the two classical turning points,

(23) $$\Delta u=x_{+}-x_{-}=2{\left(\frac{2mE_{0}}{\beta +\alpha }\right)}^{1/4}.$$

Accordingly, if Δu≈σ(t), we obtain that Δu(1.5)≈4. That is, by the time that it takes the system to pass through its initial position for the first time, the corresponding distribution has already covered nearly the whole available space, thus making difficult to detect subsequent recurrences, as it can be noticed in Figure 2a,d. Nonetheless, due to interference, the phase space cannot be homogeneously covered, as it happens with the swarms of trajectories, due to ergodicity. Hence, it can be seen that the quantum entropies display a series of fluctuations that are absent in the classical partners, which approach the saturation limit in a smooth manner. On the contrary, at higher energies, the spreading times are shorter than the orbit half periods, and hence more recurrences can be observed, which has nothing to do with interference, as the classical system also exhibits such recurrences. Thus, this can be regarded as a purely classical effect, which is enhanced as $E_{0}$ becomes larger. As it is seen in Figure 2c,f, for $E_{0}=150$, these tiny oscillations associated with the classical periodic orbit extend for a longer time. In this case, only after t≈40, the effects due to interference, associated with the second (coherence) term of the Moyal bracket, start being noticeable, particularly in the von Neumann entropy—note that these effects are more apparent in the von Neumann entropy than in the linear entropy due to the third term in Equation (11). Roughly speaking, it can be seen that the lifetime of the oscillatory part of both entropies doubles each time the energy increases in one order of magnitude, which is also the trend found with the spreading time: t(1.5)≈4, t(15)≈7.3, and t(150)≈13.1.

In the case of an underlying classically chaotic dynamics, when α=1 (see Figure 3), both the periods of the classical orbits and the spreading times become shorter, which basically turns into a fast suppression of the initial oscillatory behavior of the entropies. Interference terms are thus more prominent in the quantum entropies, particularly at low energies, when the number of eigenstates involved is relatively low, while the classical counterparts quickly reach the saturation regime (the trajectories tend to cover the whole phase space). As the energy increases, though, the fluctuations associated with interference become faster and with a smaller amplitude, which makes the quantum entropies to display a sort of saturation regime similar to the classical one. Indeed, it can be noticed that the difference between the quantum and classical linear entropies becomes negligible, with their saturation value nearly reaching the maximum. This indicates a high effective production of entanglement between *X* and *Y*, which eventually leads to maximal mixing in both subsystems’ states. Regarding the von Neumann entropies, the trend is analogous to the linear counterparts, with the relative distance between the quantum and classical average saturation values, $|S_{V}^{\mathrm{cl}}-S_{V}|/S_{V}^{\mathrm{cl}}$, becoming smaller.

It is also worth emphasizing that, due to the range of energies considered, it is possible to detect a dynamical crossover concerning the generation of entanglement in the reference system. Comparing Figure 2 and Figure 3, particularly the plots of the von Neumann entropies (although the linear entropies also show the same trend), it is readily noticed that at low energies the production of entanglement is more effective in the regular regime than in the chaotic one, both classically and quantum mechanically. Observe the asymptotic trends, that is, the maximum for the classical dynamics, and the average for the quantum mechanical one. These values are lower in the chaotic regime than in the regular one. At intermediate energies, both dynamics are similar, although the distance between the quantum and classical asymptotic trends is larger in the chaotic case than in the regular one. However, at high energies, we observe a crossover; the production of entanglement becomes more effective in the chaotic case than in the regular one. This is a counter-intuitive effect, against a common statement that underlying classically chaotic dynamics should generate entanglement more efficiently, but that confirms analogous results found in many-body interactions at different thermal regimes (and hence different dynamical behaviors) [59].

**Figure 4:**
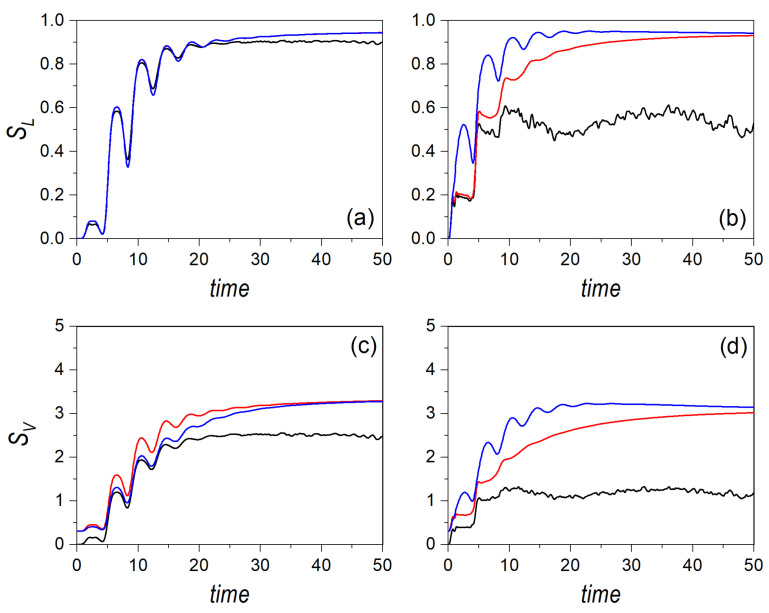
Linear entropy (upper row panels) and von Neumann entropy (lower row panels) for an initial distribution associated with a single Gaussian wave packet moving along the channel at an energy $E_{0}=15$, and affected by regularity (**a**,**c**) and chaos (**b**,**d**) conditions. In all panels, the black line denotes the quantum results, the blue line represents classical motion initially oriented along the *x*-direction, and the red line classical motion initially directed along the *y*-direction.

In order to further explore these aspects, let us now consider the case where the Gaussian distribution is launched, again, from the center of the potential, but directed along one of the transverse directions, either the *x*-direction, initially centering the Gaussian on $\left(0,0,\sqrt{2mE_{0}},0\right)$, or the *y*-direction, considering it on the alternative phase-space point $\left(0,0,0,\sqrt{2mE_{0}}\right)$. The corresponding linear and von Neumann entropies are respectively displayed in the upper and lower row panels of Figure 4 for $E_{0}=15$. Furthermore, in order to better evaluate the effects of the coupling strength, α, the results for the regular and the chaotic cases are shown, respectively, in panels (a/d) and (b/c). As it can be noticed, the quantum entropies do not discriminate the initial direction of the motion, due to the symmetry of the two selected directions. That is, it does not matter who is initially at rest, either *Y* or *X*, assigning all the initial energy, $E_{0}$, to the other subsystem. As mentioned above, in Section 2, because the joint state describing both systems is pure, both entropy measures render the same result for the two subsystems, and hence a symmetric exchange of the momentum does not produce any change in the respective entropies. The same, however, is not true in the classical case, where we can observe major differences between both directions until the entropies reach the saturation regime, regardless of the dynamical regime considered. This is consistent with the fact that the trajectories initially started with some momentum along the *x*-direction must, somehow, manifest the oscillatory behavior along this direction, with small deviations towards the *y*-direction, as $p_{y,0}=0$. In fact, following the above procedure to determine the half-period, we find that

(24) $$\tau_{1/2}^{\prime }\left(E_{0}\right)=\frac{1}{\sqrt{2mE_{0}}}\int_{x_{-}^{\prime }}^{x_{+}^{\prime }}\frac{dx}{\sqrt{1-\gamma^{\prime }x^{4}}}\approx 2.62{\left(m^{2}\beta E_{0}\right)}^{-1/4},$$

where $x_{\pm }^{\prime }=\pm \gamma^{\prime -1/4}$ denote the actual turning points of the periodic orbit along the *x*-direction, with $\gamma^{\prime }=\beta /4E_{0}$. As, now, there is no dependence on the coupling strength α, the same half-period, $\tau_{1/2}^{\prime }\left(15\right)\approx 4.21$, is obtained both in the regular and in the chaotic dynamics. This is clearly seen in all panels of Figure 4 by inspecting the blue line during the first stages of the volution (up to t≈20).

There are, though, important differences between the regular and chaotic dynamics, in particular, the observation of oscillatory motion also along the *y*-direction in the former and its absence (but with presence of kind of steps of the same duration) in the latter. This lack in the chaotic regime arises because the interaction potential acquires the shape of two crossed channels (see Figure 1b), which prevents the motion along the corresponding perpendicular direction (here, for $p_{x,0}=0$, along the *x*-direction) before the swarm of trajectories has smeared out across the available phase space. On the contrary, in the regular regime, the seemingly squared-billiard shape displayed by the interaction potential (see Figure 1a) allows an effective energy transfer from one degree of freedom to the other, as in an oscillator. When these two behaviors are compared with the quantum result, we find that the latter resembles the case of motion along the *x*-direction in the regular regime, while it draws closer to the case of motion along the *y*-direction in the chaotic regime. This has to do with the capability of the Gaussian distribution to spread across the potential well, which is more efficient in the regular case than in the chaotic one, due to the same reason mentioned above regarding the diffusion of classical trajectories.

**Figure 5:**
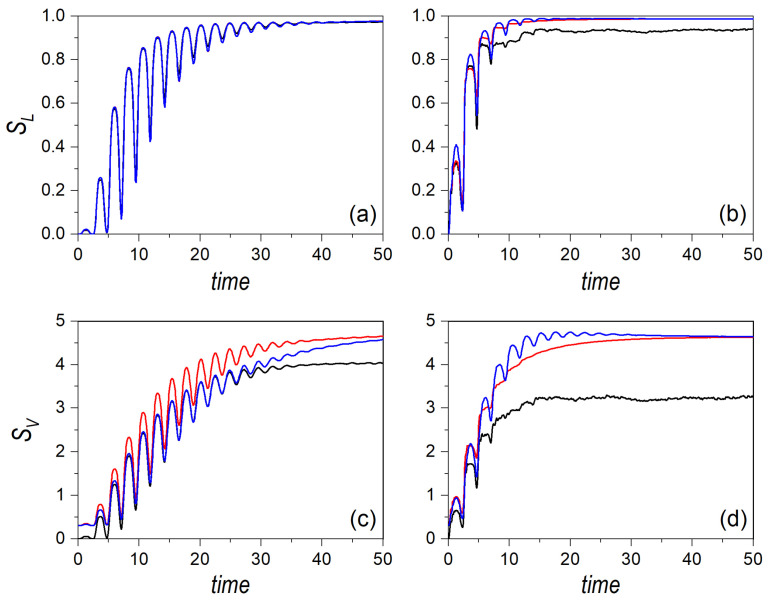
Linear entropy (upper row panels) and von Neumann entropy (lower row panels) for an initial distribution associated with a single Gaussian wave packet moving along the channel at an energy $E_{0}=150$, and affected by regularity (**a**,**c**) and chaos (**b**,**d**) conditions. In all panels, the black line denotes the quantum results, the blue line represents classical motion initially oriented along the *x*-direction, and the red line classical motion initially directed along the *y*-direction.

That situation changes if higher energies are considered, as is shown in Figure 5, for $E_{0}=150$, and where each panel represents the same quantities and conditions as in Figure 4. In this case, the half-period reduces to $\tau_{1/2}^{\prime }\left(150\right)\approx 2.37$, which allows us to observe a larger number of recurrences in the entropies. This effect is even more remarkable in the chaotic case, as, as seen above for $E_{0}=15$, such oscillatory behavior is absent in the same regime. Furthermore, unlike the distribution moving along the diagonal, here the production of entanglement is more efficient in the regular case, although the difference between the corresponding entropies becomes smaller as $E_{0}$ increases, which is consistent with the previous results. Comparing Figure 4 and Figure 5, a clear crossover trend is also apparent, as before, although it will take place at higher energies. This can be associated with the fact that, in this case, in the chaotic regime, the motion is confined within a channel for a longer time (that is, it takes longer to spread out within the well), thus displaying a lower rate of ergodicity. This is confirmed by the fact that the (asymptotic) maximum value of the entropy is lower than in the case of diagonal motion analyzed above (compare Figure 4 and Figure 5 with the analogous cases in Figure 1 and Figure 2). Thus, we can conclude that the crossover is a general trend, although, like the Lyapunov exponent in a classical dynamical system, it depends on the specific phase-space point where the quantum distribution is launched from, as well as the energy $E_{0}$ at which this is done.

As we will see next, this dynamical analysis already renders some interesting insights to understand the behaviors displayed by cat states and Bell-type entangled states, as well as the suitability of the classical counterparts here defined.

### 3.3. Cat State

Gaussian states are known for having a direct classical counterpart, although we have also seen above that there are important dynamical differences. A natural question that now arises is whether similar trends and agreements can also be found beyond these particular states. Thus, let us consider the non-classical case of an initial coherent superposition for *X*, consisting of two Gaussian wave packets centered at the phase-space points $\left(x_{1},p_{x,1}\right)$ and $\left(x_{2},p_{x,2}\right)$, coupled to *Y*, which is still described by a single Gaussian centered at $\left(y_{0},p_{y,0}\right)$ (as described in Section 3.2). The joint state reads as

(25) $$\Psi \left(x,y\right)=\frac{A}{\sqrt{2}}{\left(\frac{1}{2\pi \sigma^{2}}\right)}^{1/2}\left[e^{-{\left(x-x_{1}\right)}^{2}/4\sigma^{2}+ip_{x,1}\;\;x/\hbar }+e^{-{\left(x-x_{2}\right)}^{2}/4\sigma^{2}+ip_{x,2}\;\;x/\hbar }\right]e^{-{\left(y-y_{0}\right)}^{2}/4\sigma^{2}+ip_{y,0}\;\;y/\hbar },$$

with

(26) $$A^{-1}=\sqrt{1+e^{-{\left(\Delta x\right)}^{2}/8\sigma^{2}-\sigma^{2}{\left(\Delta p\right)}^{2}/2\hbar^{2}}\cos\left(\Delta p_{x}\bar{x}/\hbar \right)},$$

which amounts to 1 if the distance in phase space between the two wave packets is large enough, and where $\bar{x}=\left(x_{1}+x_{2}\right)/2$, $\Delta x=x_{1}-x_{2}$, and $\Delta p_{x}=p_{x,1}-p_{x,2}$. The corresponding Wigner distribution function is given by

(27) $$\begin{array}{ccc} \rho_{W}\left(x,y,p_{x},p_{y}\right)\;\;\;\; & = & \;\;\;\;\frac{A^{2}}{2}{\left(\frac{1}{\pi \hbar }\right)}^{2}\;\{e^{-{\left(x-x_{1}\right)}^{2}/2\sigma^{2}-2\sigma^{2}{\left(p_{x}-p_{x,1}\right)}^{2}/\hbar^{2}}+e^{-{\left(x-x_{2}\right)}^{2}/2\sigma^{2}-2\sigma^{2}{\left(p_{x}-p_{x,2}\right)}^{2}/\hbar^{2}}\\ \;\;\;\; & & \;\;\;\;\;+2e^{-{\left(x-\bar{x}\right)}^{2}/2\sigma^{2}-2\sigma^{2}{\left(p_{x}-{\bar{p}}_{x}\right)}^{2}/\hbar^{2}}\cos\left[\frac{\Delta p_{x}x-\left(p_{x}-{\bar{p}}_{x}\right)\Delta x}{\hbar }\right]\},\\ \;\;\;\; & & \;\;\;\;\;\times e^{-{\left(y-y_{0}\right)}^{2}/2\sigma^{2}-2\sigma^{2}{\left(p_{y}-p_{y,0}\right)}^{2}/\hbar^{2}}, \end{array}$$

with ${\bar{p}}_{x}=\left(p_{x,1}+p_{x,2}\right)/2$. Again, due to the separability of this joint state, integrating over the *Y*-system variables has no influence on the reduced Wigner distribution function for *X*, which reads as

(28) $$\begin{array}{ccc} {\tilde{\rho }}_{W}\left(x,p_{x}\right)\;\;\;\; & = & \;\;\;\;\frac{A^{2}}{2\pi \hbar }\;\{e^{-{\left(x-x_{1}\right)}^{2}/2\sigma^{2}-2\sigma^{2}{\left(p_{x}-p_{x,1}\right)}^{2}/\hbar^{2}}+e^{-{\left(x-x_{2}\right)}^{2}/2\sigma^{2}-2\sigma^{2}{\left(p_{x}-p_{x,2}\right)}^{2}/\hbar^{2}}\\ \;\;\;\; & & \;\;\;\;+2e^{-{\left(x-\bar{x}\right)}^{2}/2\sigma^{2}-2\sigma^{2}{\left(p_{x}-{\bar{p}}_{x}\right)}^{2}/\hbar^{2}}\cos\left[\frac{\Delta p_{x}x-\left(p_{x}-{\bar{p}}_{x}\right)\Delta x}{\hbar }\right]\}. \end{array}$$

Unlike (17), note that now the Wigner distribution function (27) is negative definite on certain phase-space regions (actually in the subspace corresponding to the subsystem *X*, as seen in (28)), which is a distinctive trait of the mutual coherence between the two wave packets. This negativity makes the correspondence with a classical counterpart unclear. A way to proceed could be assigning a negative flux to classical initial conditions (trajectories) picked up within the negative regions. However, the physical meaning of classical density distribution would get lost, and the Shannon entropy could not be computed in the way described above. Instead, another procedure has been chosen. In (28), there are three terms. Two of them correspond to having the system localized either around the phase-space points ($x_{1},p_{1}$) and ($x_{2},p_{2}$), according to identical Gaussian distributions. The third term, the interference one associated with the negativity of the Wigner distribution function, is a cosine modulated by a Gaussian identical to the two previous ones, but centered around the mean values $\bar{x}$ and ${\bar{p}}_{x}$. As this is the contribution that makes the Wigner distribution function to be non-classical, let us simply remove it and analyze its dynamical behavior in terms of the linear and von Neumann entropy measures. Let us thus assume that the “classical” cat state is a bare sum of localized Gaussian distributions, such that

(29) $$\begin{array}{ccc} \rho_{\mathrm{cl}}\left(x,y,p_{x},p_{y}\right)\;\;\;\; & = & \;\;\;\;\frac{1}{2}{\left(\frac{1}{\pi \hbar }\right)}^{2}\;\left[e^{-{\left(x-x_{1}\right)}^{2}/2\sigma^{2}-2\sigma^{2}{\left(p_{x}-p_{x,1}\right)}^{2}/\hbar^{2}}+e^{-{\left(x-x_{2}\right)}^{2}/2\sigma^{2}-2\sigma^{2}{\left(p_{x}-p_{x,2}\right)}^{2}/\hbar^{2}}\right]\\ \;\;\;\; & & \;\;\;\;\;\;\;\times e^{-{\left(y-y_{0}\right)}^{2}/2\sigma^{2}-2\sigma^{2}{\left(p_{y}-p_{y,0}\right)}^{2}/\hbar^{2}}, \end{array}$$

while the reduced density distribution is simply given by

(30) $${\tilde{\rho }}_{\mathrm{cl}}\left(x,p_{x}\right)=\frac{1}{2\pi \hbar }\;\left[e^{-{\left(x-x_{1}\right)}^{2}/2\sigma^{2}-2\sigma^{2}{\left(p_{x}-p_{x,1}\right)}^{2}/\hbar^{2}}+e^{-{\left(x-x_{2}\right)}^{2}/2\sigma^{2}-2\sigma^{2}{\left(p_{x}-p_{x,2}\right)}^{2}/\hbar^{2}}\right],$$

which lacks the prefactor $A^{2}$ precisely because there is no interference term.

Unlike the single Gaussian distribution, now none of the classical entropies are going to equal the quantum mechanical counterparts for these cat states. This time the linear entropy reads as

(31) $$S_{L}^{\mathrm{cl}}=\frac{1}{2}\left[1-e^{-{\left(\Delta x\right)}^{2}/4\sigma^{2}-\sigma^{2}{\left(\Delta p_{x}\right)}^{2}/\hbar^{2}}\right].$$

Thus, if the distance in phase space is relatively large, this quantity amounts to $S_{L}^{\mathrm{cl}}\approx 0.5$. The fact that this quantity does not vanish can be interpreted as a consequence of having the classical distribution delocalized between two different regions in phase space. This will also have an influence on the classical von Neumann entropy, which can be written as

(32) $$\begin{array}{ccc} S_{V}^{\mathrm{cl}}\;\;\;\; & = & \;\;\;\;-\int \frac{1}{2}\;\left({\tilde{\rho }}_{\mathrm{cl},1}+{\tilde{\rho }}_{\mathrm{cl},2}\right)\ln\left[\frac{1}{2}\;\left({\tilde{\rho }}_{\mathrm{cl},1}+{\tilde{\rho }}_{\mathrm{cl},2}\right)\right]\\ \;\;\;\; & ≃ & \;\;\;\;-\frac{1}{2}\int {\tilde{\rho }}_{\mathrm{cl},1}\ln{\tilde{\rho }}_{\mathrm{cl},1}-\frac{1}{2}\int {\tilde{\rho }}_{\mathrm{cl},2}\ln{\tilde{\rho }}_{\mathrm{cl},2}+\frac{1}{2}\int \left({\tilde{\rho }}_{\mathrm{cl},1}+{\tilde{\rho }}_{\mathrm{cl},2}\right)\ln2, \end{array}$$

if the reduced Wigner distribution function (30) is recast as

(33) $${\tilde{\rho }}_{\mathrm{cl}}\left(x,p_{x}\right)=\frac{1}{2}\left[{\tilde{\rho }}_{\mathrm{cl},1}\left(x,p_{x}\right)+{\tilde{\rho }}_{\mathrm{cl},2}\left(x,p_{x}\right)\right]$$

and the assumption of a relatively long distance in phase space between the two Gaussians is again considered. Now, given the symmetry of the problem, the integrals for both distributions render the same result, and hence the above expression can be written, without loss of generality, as

(34) $$S_{V}^{\mathrm{cl}}\approx -\int {\tilde{\rho }}_{\mathrm{cl},1}\ln{\tilde{\rho }}_{\mathrm{cl},1}+\ln2\int {\tilde{\rho }}_{\mathrm{cl},1}=1,$$

where (20) has been used in the last equality. It is thus seen that the von Neumann entropy for the classical distribution does not simply doubles the result for a single Gaussian, but, due to the delocalization, the lower bound amounts to 1.

The above differences between the classical and quantum entropies, once the interference term has been removed in the quantum distribution in order to define the classical one, already provide us with some clues as to why quantum entropies always remain lower than classical ones. This was an aspect already mentioned in the case of the Gaussian distributions, namely, that quantum distributions optimize the compression of phase-space information by including such an interference term; however, this is not obvious, because there was no such term included. Only as time proceeds and the interference starts developing does this become clearer. On the contrary, because the two subsystems become more entangled with time, the systems’ interference traits are gradually suppressed, which eventually leads the quantum and classical linear entropies to draw closer, and the von Neumann ones to reduce the distance between corresponding quantum and classical saturation (asymptotic) values. On the other hand, the choice for the classical partner of the quantum cat here will also be clearer in next section, when we will see that the same functional form also arises in the case of the entangled state. This “classical” partner for the entangled state emerges, precisely, after the coupling (entanglement) with the environment washes out the interference term. This thus makes clear why the same delocalized classical distribution serves to set a comparison with both the cat state (quantum delocalization before undergoing decoherence) and the Bell-type entangled state (quantum delocalization after undergoing decoherence).

**Figure 6:**
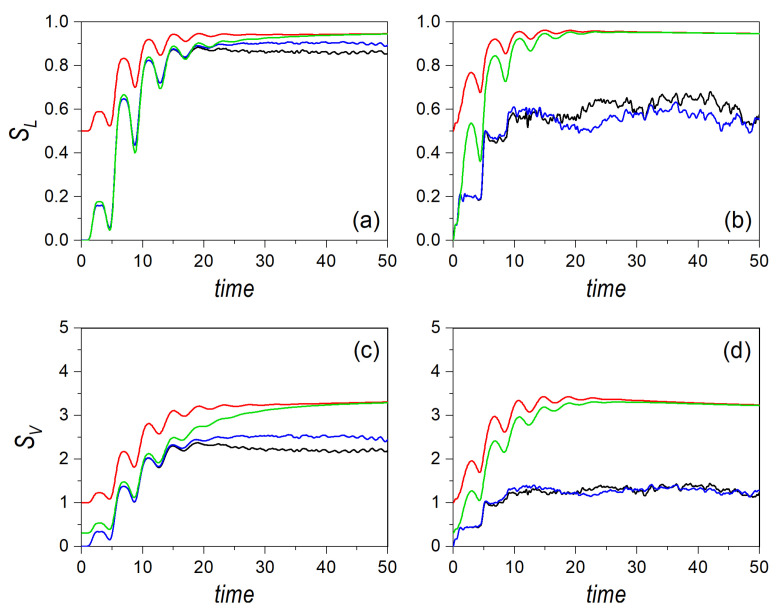
Linear entropy (upper row panels) and von Neumann entropy (lower row panels) for an initial distribution associated with a cat state aligned along the *x*-channel, with an energy $E_{0}=15$, and affected by regularity conditions (**a**,**c**) and chaos conditions (**b**,**d**). In all panels, black line denotes the quantum results, while the red line refers to the classical ones. To compare with, the quantum and classical results for a single Gaussian distribution, with the initial at $p_{2}$ (see text for details), are also included, and denoted, respectively, with the blue and green lines.

Such a trend is better understood by considering the dynamical evolution of the entropies. Thus, consider a cat state formed by two Gaussian wave packets initially localized at the phase space points $p_{1}=\left(-x_{0},0,\sqrt{2mE_{0}^{\prime }},0\right)$ and $p_{2}=\left(x_{0},0,-\sqrt{2mE_{0}^{\prime }},0\right)$, where $x_{0}=2.5$ and $E_{0}^{\prime }=E_{0}-\beta x_{0}^{4}/4$, with $E_{0}=15$. This initial condition ensures a negligible overlapping. The time evolution of the linear and von Neumann entropies are displayed, respectively, in the upper and lower row panels of Figure 6, in the left column for a regular dynamics (α=0.03) and in the right column for the chaotic one (α=1). Furthermore, to compare with, and hence to determine the relationship between the single, localized Gaussian state and the delocalized cat state, the corresponding results for a single Gaussian centered at $p_{2}$ are also shown. When comparing all these cases, several interesting features are worth stressing. First of all, in all cases, except for some minor deviations, the quantum results for the cat state and the single Gaussian distribution are identical; actually, in the chaotic regime, note that the results mimic those of the Gaussian moving along the transverse channel (see Figure 4). This means that, quantum mechanically, the entropy measures do not make any distinction between having a localized distribution in phase space or two separate distributions coherently connected by the the interference term. Classically, there is an initial difference, as was already discussed above, but, as time proceeds, both distributions, localized and delocalized, approach the same asymtotic value due to ergodicity. Furthermore, we can observe that the initial increase stage is characterized by the same oscillatory behavior discussed in the previous section. Notice that, although the entropies for the classical cat-state distribution start with non-vanishing values, each Gaussian contributes independently due to their incoherence (the trajectories associated with one of these Gaussians will display mirror symmetry with respect to the trajectories related to the other Gaussian, thus rendering the same oscillation period and behavior).

### 3.4. Bell-Type Maximally Entangled State

Finally, let us consider a Bell-type or maximally entangled state consisting of two delocalized Gaussians for each subsystem, with centers $\left(x_{1},p_{x,1}\right)$ and $\left(x_{2},p_{x,2}\right)$ for *X*, and $\left(y_{1},p_{y,1}\right)$ and $\left(y_{2},p_{y,2}\right)$ for *Y*, such that $\left(y_{1},p_{y,1}\right)=\left(x_{2},p_{x,2}\right)$ and $\left(y_{2},p_{y,2}\right)=\left(x_{1},p_{x,1}\right)$:

(35) $$\begin{array}{ccc} \Psi (x,y)\; & = & \;\frac{A}{\sqrt{2}}{\left(\frac{1}{2\pi \sigma^{2}}\right)}^{1/2}\;[e^{-{\left(x-x_{1}\right)}^{2}/4\sigma^{2}+ip_{x,1}x/\hbar }e^{-{\left(y-y_{1}\right)}^{2}/4\sigma^{2}+ip_{y,1}y/\hbar }\\ \;\;\;\; & & \;\;\;\;\;+e^{-{\left(x-x_{2}\right)}^{2}/4\sigma^{2}+ip_{x,2}x/\hbar }e^{-{\left(y-y_{2}\right)}^{2}/4\sigma^{2}+ip_{y,2}y/\hbar }], \end{array}$$

where

(36) $$A^{-1}=\sqrt{1+e^{-{\left(\Delta x\right)}^{2}/8\sigma^{2}-\sigma^{2}{\left(\Delta p_{x}\right)}^{2}/2\hbar^{2}}e^{-{\left(\Delta y\right)}^{2}/8\sigma^{2}-\sigma^{2}{\left(\Delta p_{y}\right)}^{2}/2\hbar^{2}}\cos\left(\frac{\Delta p_{x}\bar{x}+\Delta p_{y}\bar{y}}{\hbar }\right)},$$

with $\bar{x}$, Δx, and $\Delta p_{x}$, and their homologs for *Y*, defined as before. The associated Wigner distribution function is

(37) $$\begin{array}{ccc} \rho_{W}\left(x,y,p_{x},p_{y}\right)\;\;\;\; & = & \;\;\;\;\frac{A^{2}}{2}{\left(\frac{1}{\pi \hbar }\right)}^{2}\{e^{-{\left(x-x_{1}\right)}^{2}/2\sigma^{2}-2\sigma^{2}{\left(p_{x}-p_{x,1}\right)}^{2}/\hbar^{2}}e^{-{\left(y-y_{1}\right)}^{2}/2\sigma^{2}-2\sigma^{2}{\left(p_{y}-p_{y,1}\right)}^{2}/\hbar^{2}}\\ \;\;\;\; & & \;\;\;\;\;+e^{-{\left(x-x_{2}\right)}^{2}/2\sigma^{2}-2\sigma^{2}{\left(p_{x}-p_{x,2}\right)}^{2}/\hbar^{2}}e^{-{\left(y-y_{2}\right)}^{2}/2\sigma^{2}-2\sigma^{2}{\left(p_{y}-p_{y,2}\right)}^{2}/\hbar^{2}}\\ \;\;\;\; & & \;\;\;\;\;+2e^{-{\left(x-\bar{x}\right)}^{2}/2\sigma^{2}-2\sigma^{2}{\left(p_{x}-\bar{p}\right)}^{2}/\hbar^{2}}e^{-{\left(y-\bar{y}\right)}^{2}/2\sigma^{2}-2\sigma^{2}{\left(p_{y}-\bar{y}\right)}^{2}/\hbar^{2}}\\ \;\;\;\; & & \;\;\;\;\;\;\times \cos\left[\frac{\Delta p_{x}\;\;x+\Delta p_{y}\;\;y-\left(p_{x}-{\bar{p}}_{x}\right)\Delta x-\left(p_{y}-{\bar{p}}_{y}\right)\Delta y}{\hbar }\right]\}, \end{array}$$

which also contains an interference term that generates negative definite phase-space regions, although now such regions are not embedded within a particular subspace, but affect the full four-dimensional phase space. Accordingly, because (37) is not factorizable, this time the reduced Wigner distribution function for *X* is going to depend on parameters associated with *Y*:

(38) $$\begin{array}{ccc} {\tilde{\rho }}_{W}\left(x,p_{x}\right)\;\;\;\; & = & \;\;\;\;\frac{A^{2}}{2\pi \hbar }\;\{e^{-{\left(x-x_{1}\right)}^{2}/2\sigma^{2}-2\sigma^{2}{\left(p_{x}-p_{1}^{x}\right)}^{2}/\hbar^{2}}+e^{-{\left(x-x_{2}\right)}^{2}/2\sigma^{2}-2\sigma^{2}{\left(p_{x}-p_{2}^{x}\right)}^{2}/\hbar^{2}}\\ \;\;\;\; & & \;\;\;\;\;\;+2e^{-{\left(\Delta y\right)}^{2}/8\sigma^{2}-\sigma^{2}{\left(\Delta p_{y}\right)}^{2}/2\hbar^{2}}e^{-{\left(x-\bar{x}\right)}^{2}/2\sigma^{2}-2\sigma^{2}{\left(p_{x}-\bar{p}\right)}^{2}/\hbar^{2}}\\ \;\;\;\; & & \;\;\;\;\;\;\times \cos\left[\frac{\Delta p_{y}\;\;\bar{y}+\Delta p_{x}\;\;x-\left(p_{x}-{\bar{p}}_{x}\right)\Delta x}{\hbar }\right]\}. \end{array}$$

Note that this explicit dependence on *Y* in the interference term implies that two separate phase-space points in the *Y*-subspace are enough to cancel out this term, eventually leading to the classical distribution considered for the cat state, Equation (29). Actually, if we choose as the classical Wigner distribution function here the one given by (37), but without the crossed interference (coherence) term, i.e.,

(39) $$\begin{array}{ccc} \rho_{\mathrm{cl}}\left(x,y,p_{x},p_{y}\right)\;\;\;\; & = & \;\;\;\;\frac{1}{2}{\left(\frac{1}{\pi \hbar }\right)}^{2}\left[e^{-{\left(x-x_{1}\right)}^{2}/2\sigma^{2}-2\sigma^{2}{\left(p_{x}-p_{x,1}^{x}\right)}^{2}/\hbar^{2}}e^{-{\left(y-y_{1}\right)}^{2}/2\sigma^{2}-2\sigma^{2}{\left(p_{y}-p_{y,1}^{y}\right)}^{2}/\hbar^{2}}\right.\\ \;\;\;\; & & \;\;\;\;\left.+e^{-{\left(x-x_{2}\right)}^{2}/2\sigma^{2}-2\sigma^{2}{\left(p_{x}-p_{x,2}^{x}\right)}^{2}/\hbar^{2}}e^{-{\left(y-y_{2}\right)}^{2}/2\sigma^{2}-2\sigma^{2}{\left(p_{y}-p_{y,2}^{y}\right)}^{2}/\hbar^{2}}\right], \end{array}$$

the reduced Wigner distribution is exactly the same as (29):

(40) $${\tilde{\rho }}_{\mathrm{cl}}\left(x,p_{x}\right)=\frac{1}{2\pi \hbar }\;\left\{e^{-{\left(x-x_{1}\right)}^{2}/2\sigma^{2}-2\sigma^{2}{\left(p_{x}-p_{1}\right)}^{2}/\hbar^{2}}+e^{-{\left(x-x_{2}\right)}^{2}/2\sigma^{2}-2\sigma^{2}{\left(p_{x}-p_{2}\right)}^{2}/\hbar^{2}}\right\}.$$

Therefore, in both cases, cat state and entangled state, we eventually reach the same reduced Wigner distribution function, although the joint Wigner distribution function is different, as it is a simple delocalization in the *X*-subspace for the former, while it corresponds to a exchange or swapping in the joint XY-space for the latter.

Regarding the initial value of the entropies, now we have

(41) $$\begin{array}{ccc} S_{L}\;\;\;\; & = & \;\;\;\;1-\frac{A^{4}}{2}\;\left[g_{x}^{2}+g_{y}^{2}-g_{x}^{2}g_{y}^{2}-1+2{\left(1+g_{x}g_{y}\cos\phi xy\right)}^{2}\right], \end{array}$$

(42) $$\begin{array}{ccc} S_{L}^{\mathrm{cl}}\;\;\;\; & = & \;\;\;\;\frac{1}{2}\left(1-g_{x}^{2}\right), \end{array}$$

with

$$\begin{array}{ccc} g_{x}\;\;\;\; & = & \;\;\;\;e^{-{\left(\Delta x\right)}^{2}/8\sigma^{2}-\sigma^{2}{\left(\Delta p_{x}\right)}^{2}/2\hbar^{2}},\\ g_{y}\;\;\;\; & = & \;\;\;\;e^{-{\left(\Delta y\right)}^{2}/8\sigma^{2}-\sigma^{2}{\left(\Delta p_{y}\right)}^{2}/2\hbar^{2}},\\ \phi_{xy}\;\;\;\; & = & \;\;\;\;\left(\Delta p_{y}\bar{y}+\Delta p_{x}\bar{x}\right)/\hbar . \end{array}$$

In the case that the phase-space points are far apart, the two values approach the same lower bound, $S_{L}=S_{L}^{\mathrm{cl}}=0.5$. However, we also find that, even if this separation condition only satisfies for the *Y* phase-space points, both entropies coincide, although to the lower bound

(43) $$S_{L}=S_{L}^{\mathrm{cl}}=\frac{1}{2}\left(1-g_{x}^{2}\right),$$

which stresses the fact that decoherence due to the entanglement with *Y* leads to a delocalized classical-type state. Indeed, it is also observed that, as the phase-space points around which the Gaussian distributions are centered draw closer, and hence the delocalization becomes smaller, this lower bound for the linear entropy gets smaller. In the limit of full localization (both Gaussian distributions centered around the same phase-space point), both linear entropies vanish, which corresponds to the case analyzed in Section 3.2. On the other hand, with respect to the von Neumann entropy, in this case neither the quantum entropy nor the classical one are zero. The value for the classical one was given above, $S_{V}^{\mathrm{cl}}=1$, while the quantum value can readily be obtained by directly applying the second equality of Equation (10) to this case, which renders the well-known result for maximally entangled bipartite systems, $S_{V}=\ln2\approx 0.693$.

**Figure 7:**
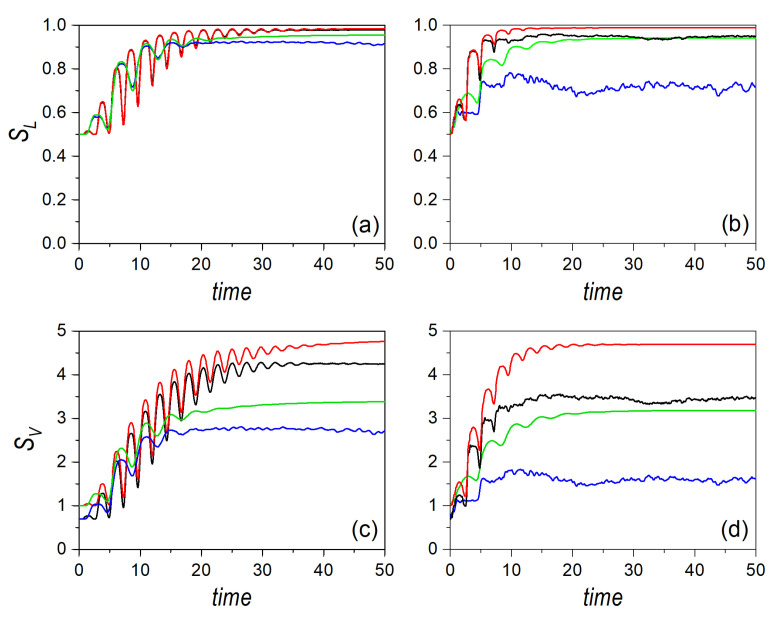
Linear entropy (upper row) and von Neumann entropy (lower row) for an initial distribution associated with a Bell-type entangled state for two different values of the energy, and affected by regularity conditions (**a**,**c**) and chaos conditions (**b**,**d**). Results for $E_{0}=15$ are denoted with the blue and green lines, respectively, for the quantum and the classical dynamical regimes. Results for $E_{0}=150$ are denoted with the black and red lines, respectively, for the quantum and the classical dynamical regimes.

Let us now analyze the behavior displayed by the corresponding phase-space distributions. To this end, the maximally entangled state selected is specified by positioning the Gaussians at the phase-space points $p_{1}=\left(x_{0},0,p_{x,0},0\right)$ and $p_{2}=\left(0,y_{0},0,p_{y,0}\right)$, where $x_{0}=y_{0}=2.5$ and $p_{x,0}=p_{y,0}=-\sqrt{2mE_{0}^{\prime }}$, with $E_{0}^{\prime }=E_{0}-\beta x_{0}^{4}/4$. In this case, in order to better understand the approach to the classical limit by increasing the total energy, we have considered $E_{0}=15$ and $E_{0}=150$. Note that the initial configuration denotes symmetry with respect to the y=x axis, while the initial motion of the wave packets takes place along the *x*- and *y*-channels, respectively, which will render some similarities to former results already discussed. The time evolution for the quantum and classical entropies corresponding to a maximally entangled state are displayed in Figure 7, the linear entropies in the upper panels and the von Neumann ones in the lower panels; in the left column for the regular regime, and in the right column for the chaotic one. In both cases, contrary what one might expect, as we also observed with the cat state, the behavior here, for both energies, is pretty similar to the behavior found for analogous conditions (i.e., initial in-channel motion) in the case of a single Gaussian distribution. Of course, due to the seemingly incoherent addition of the two quantum Gaussian distributions (led by the decoherence induced by the entanglement between the two subsystems), the initial values are different compared to those of the initial localized Gaussian, and in compliance with the values analytically determined and discussed above. However, as time proceeds, both the oscillatory initial increase and the final asymptotic saturation regime are pretty similar to the results previously found for the Gaussian distributions, even regarding the separation between quantum and classical counterparts.

## 4. Discussion

As explained in the introductory section, the main goal of the present work was to explore whether there is any sort of quantum–classical correspondence in the case of the entropy measures commonly used to quantify entanglement in (joint) pure bipartite systems, namely, the linear and the von Neumann entropies. As entanglement is a unique quantum feature, in principle, one should assume that such correspondence does not take place. On the other hand, we also know that, as decoherence effects become stronger due to the entanglement between the two parties, the manifestation of quantum–classical correspondence should be more apparent. Thus, with the purpose to compare on equal footing, and hence to determine whether any quantum–classical correspondence can be established, two coupled continuous-variable bipartite systems have been considered, consisting of two one-dimensional quartic oscillators nonlinearly coupled by means of a biquadratic term, which has been shown to display either regular or chaotic dynamics depending on the coupling strength. The entropy measures have then been recast in terms phase-space distributions, which are a common ground for comparison purposes to both quantum and classical mechanics, even though there are still certain differences and subtleties associated with the coherence properties of quantum systems (e.g., the negativity of the Wigner distribution function, which is lacking in classical phase-space density distributions). Furthermore, it has also been necessary to define classical analogs of the initial Wigner distribution functions, which has been achieved by simply removing the coherent part in the latter, i.e., suppressing the partly negative-definite interference terms.

With those ingredients, the time evolution displayed by the linear and von Neumann entropies associated with three particular types of initial states have been analyzed:

- Gaussian states, which have been chosen, because these factorizable, localized states keep a very close connection to classical distributions of the same kind, thus allowing us to better understand how the development and presence of interference is at the origin of the long term differences between the quantum and classical asymptotic entropy values. In the short and medium terms, however, both the quantum and classical results are mainly governed by the oscillations of the system inside the potential in a similar manner. Nonetheless, in all cases, the classical values constitute, at each time, an upper bound for the quantum ones, which draw closer to the former in the case of the linear entropy, but present a gap in the case of the von Neumann entropy. This gap becomes smaller as the total energy becomes larger (the system becomes more classical), although there is not a clear trend denoting that it vanishes, as in the case of the linear entropy. Hence, the gap can be associated with the coherence characterizing the quantum system, which allows it to reduce its entropy unlike the classical system, where its incoherence does not allow lower values of the entropy. This is something that we also observe in the initial values of the von Neumann entropy, which is zero in the quantum case (minimum amount of information to specify the state) and nonzero for the classical. Note that, although the initial state is the same in both cases (an everywhere positive-definite Gaussian distribution), the appearance of the third term in the last equality of (11), which is responsible for interference-related contributions (hence leading to the inequality ${\left(\ln\tilde{\rho }\right)}_{W}\neq \ln{\tilde{\rho }}_{W}$), sets a major difference. This is, nonetheless, different from the appearance of the HOC term in (2), which is responsible for the development of interference-type features in relation to the interaction potential (otherwise, the dynamical evolution would be identical).
- Cat states (for the reference system), which has been selected because, regardless of factorizability, they constitute a first approach to delocalization with presence of coherence. Although the quantum system is delocalized, because the two (quantum) Gaussian amplitudes are coherently superimposed, there is a share of mutual phase information, which translates into a common intermediate region in phase space that is partly negative definite. The classical counterpart chosen is the truncated Wigner distribution function that arises when such interference term is removed. Interestingly, similar trends to those observed with the Gaussian distributions are also present here, which clearly indicate some degree of correspondence. However, because the classical distribution is delocalized, none of the entropy measures renders a vanishing value, as it does in the quantum case. This thus supports the fact that the entropy measures, apart from dynamical information (e.g., the oscillatory behavior in the short and medium term, and the saturation due to ergodicity in the long term), also contain information about the coherence of the distribution and the degree of localization. Note, in this latter regard, that the initial value for the classical linear entropy does not vanish, as it does for single Gaussian distribution.
- Bell-type entangled states, which constitute a paradigm of maximally entangled states, and that, as it has been pointed out, generate delocalization of the reference system with suppression of the interference term if the two possible phase-space locations for the environmental degree of freedom are sufficiently far from each other. In this case, the classical distribution, which is the same ansatz also associated with the cat state, looks exactly the same as the quantum Wigner distribution function. However, unlike the case of the cat state, now the suppression of the interference term by entanglement provides us with a more “classical” state, as the two density distributions that appear in phase space are incoherent (coherence is only preserved in the joint space). Yet, although we are talking about a maximally entangled state, and both the linear and the von Neumann entropies behave as expected quantum mechanically, rendering nonzero initial values, again we find a good correspondence with the classical counterparts in all cases considered. In fact, leaving aside the initial values, the trend observed in time again matches the trends obtained for the corresponding single Gaussian distributions rather well.

## 5. Concluding Remarks

In summary, in the light of the above results, we can say that there is not a major difference between the quantum and classical results in a situation where, in principle, one would assume that there is no classical analog. In fact, we conclude just the opposite, that it seems there is a nice quantum–classical correspondence. Therefore, the widespread idea that the linear and von Neumann entropies are good measures of entanglement seems to fail, as they can be fairly reproduced classically by means of simple classical analogs, obtained from the suppression of the coherence (interference) terms in the corresponding Wigner distribution functions. Indeed, at any time, it has been seen that the classical value is always above the quantum mechanical one, which could be wrongly interpreted, according to our current understanding of these entropies, as a more efficient production of entanglement from classical states than from quantum ones. After all, it has been common to regard classically chaotic regimes as more efficient in the production of entanglement than regular ones by inspecting the same kind of entropies and their time evolution (although here it has been proven that not always this is true, for there is a crossover that depends on the total energy available to the joint system). Therefore, from the results here obtained, particular confronting the simulations shown in Section 3.4 with those from Section 3.2, it seems that these entropy measures, actually, quantify the degrees of delocalization and incoherence that affect the system, which are present in both the quantum and the classical domains. Of course, in the quantum domain, because the full joint system preserves its coherence, the interaction increases the correlations between both and, hence, the entanglement, which is nothing but a coherence swapping or redistribution between both subsystems, in such a way that its effective manifestation in any of them is in the form of decoherence. In the subsystem of reference, decoherence washes out interference-related terms, thus producing a seemingly classical distribution. Yet, the preservation of coherence at a higher order (i.e., in the phase space of the complete system) makes a difference in entropy measures that emphasize such orders, such as the von Neumann entropy, which thus remains manifestly lower than its classical partner.

## Data Availability

Not applicable.
